# NESM: a network embedding method for tumor stratification by integrating multi-omics data

**DOI:** 10.1093/g3journal/jkac243

**Published:** 2022-09-19

**Authors:** Feng Li, Zhensheng Sun, Jin-Xing Liu, Junliang Shang, Lingyun Dai, Xikui Liu, Yan Li

**Affiliations:** School of Computer Science, Qufu Normal University, Rizhao 276826, China; School of Computer Science, Qufu Normal University, Rizhao 276826, China; School of Computer Science, Qufu Normal University, Rizhao 276826, China; School of Computer Science, Qufu Normal University, Rizhao 276826, China; School of Computer Science, Qufu Normal University, Rizhao 276826, China; Department of Electrical Engineering and Information Technology, Shandong University of Science and Technology, Jinan, Shandong 250031, China; Department of Electrical Engineering and Information Technology, Shandong University of Science and Technology, Jinan, Shandong 250031, China

**Keywords:** cancer subtype, multi-omics, pan-cancer, embedding network

## Abstract

Tumor stratification plays an important role in cancer diagnosis and individualized treatment. Recent developments in high-throughput sequencing technologies have produced huge amounts of multi-omics data, making it possible to stratify cancer types using multiple molecular datasets. We introduce a Network Embedding method for tumor Stratification by integrating Multi-omics data. Network Embedding method for tumor Stratification by integrating Multi-omics pregroup the samples, integrate the gene features and somatic mutation corresponding to cancer types within each group to construct patient features, and then integrate all groups to obtain comprehensive patient information. The gene features contain network topology information, because it is extracted by integrating deoxyribonucleic acid methylation, messenger ribonucleic acid expression data, and protein–protein interactions through network embedding method. On the one hand, a supervised learning method Light Gradient Boosting Machine is used to classify cancer types based on patient features. When compared with other 3 methods, Network Embedding method for tumor Stratification by integrating Multi-omics has the highest AUC in most cancer types. The average AUC for stratifying cancer types is 0.91, indicating that the patient features extracted by Network Embedding method for tumor Stratification by integrating Multi-omics are effective for tumor stratification. On the other hand, an unsupervised clustering algorithm Density-Based Spatial Clustering of Applications with Noise is utilized to divide single cancer subtypes. The vast majority of the subtypes identified by Network Embedding method for tumor Stratification by integrating Multi-omics are significantly associated with patient survival.

## Introduction

Cancer is generally due to a variety of factors under the occurrence of somatic variation, which can lead to the cell growth of abnormal regulation and the formation of abnormal lesions (Zhong *et al.* 2021). There are differences in cellular morphology and tissue structure between neoplastic and normal tissues. Benign neoplasms are often present relatively low atypia and same with the normal tissues from which they originate, while malignant neoplasms are present relatively high atypia (Liu *et al.* 2021). In recent years, the development of next-generation sequencing technologies and the development of several multicenter cancer exome/genome projects, The Cancer Genome Atlas (TCGA) and the International Cancer Genome Consortium (Chang *et al.* 2013; Jennings and Hudson 2016), provide a large amount of omics data, such as gene expression data, copy number variation, burst, and deoxyribonucleic acid (DNA) methylation data. Thus, the rapid accumulation of multi-omics tumor data has brought new opportunities and challenges to study systems biology from multilevel (Ruan *et al.* 2019).

The stratification of tumors into clinical and biological subtypes benefits precision oncology. For example, an entropy-based consensus clustering (ECC) method (Liu *et al.* 2017) for patient stratification fuses multiple base partitions into a consensus model using an entropy-based utility function. Importantly, numerous data-driven approaches for classifying cancers based on diverse clinical data have been proposed, such as multiple gene classifiers for breast cancer prognosis based on gene expression profiles (Reis-Filho and Pusztai 2011), neural network-based survival prediction for different breast cancer subtypes by combining with clinical information including tumor size and axillary lymph node status (Lundin *et al.* 1999), and skin cancer classification based on imaging data using deep neural network algorithms (Esteva *et al.* 2017).

Integrating multiplatform molecular data, such as gene expression data, miRNA expression data, and DNA methylation data, can effectively identify cancer subtypes (Liang *et al.* 2021), which has been proven to be more powerful than a single data type (Wang *et al.* 2014). Multiple strategies have posited for the integration of multiple sets of data. One strategy is to analyze each data type individually before integrating multiple sets of data (Shen *et al.* 2009; Mo *et al.* 2013), but this strategy cannot capture relationships between same-origin data. Scluster (Ge *et al.* 2017) and joint and individual variation explained (Lock *et al.* 2013) can capture the association information both between and within multiple data simultaneously, but they are sensitive to feature selection. To effectively extract shared and complementary information concealed in a variety of biological data types, more systematic and integrated methodologies are required (Zhao and Yan 2020). However, biomolecular networks contain many different layers and different organizational forms in biological systems, which have been widely used in cancer research (Leiserson *et al.* 2015; Horn *et al.* 2018; Liu *et al.* 2020; Zhang and Wang 2022). Therefore, network-based strategy is an effective method to analyze and integrate multi-omics data (Ma’ayan 2011; Zhao *et al.* 2015; Zhu *et al.* 2015; Lee *et al.* 2016).

The cancer somatic mutation spectrum can be integrated into biomolecular networks (Cheng *et al.* 2014). Cancer evolution may be influenced by somatic mutations in cancer driver genes that cause alterations in other genes. Hofree *et al.* posited a network-based stratification (NBS) method, which applies network propagation to discover cancer subtypes by gathering patients with comparable network mutations (Hofree *et al.* 2013). The hypothesis is that if the mutated genes of 2 tumors are located in similar network regions, they may be very similar. Chuang Liu *et al.* proposed a network embedding-based stratification (NES) approach for identifying clinically relevant patient categories from the somatic mutation spectrum of a large number of patients (Liu *et al.* 2021). Therefore, we can analyze each patient based on their somatic mutation spectrum and the similarities among patients to stratify tumors.

In this work, we introduce a network embedding method for tumor Stratification by integrating Multi-omics data, called NESM. NESM pregroup the samples, integrate the gene features and somatic mutation corresponding to cancer types within each group to construct patient features, and then integrate all groups to obtain comprehensive patient information. The gene features contain network topology information, because it is extracted by integrating DNA methylation, messenger ribonucleic acid (mRNA) expression data and protein–protein interactions (PPIs) through network embedding method. First, we cluster the samples with DNA methylation and mRNA expression data and calculate the Pearson correlation between genes in each cluster. Then, the gene pairs with strong correlation are preserved in PPI. Next, patient features are constructed by integrating corresponding gene features and somatic mutation profiles of cancer types. Finally, a supervised learning method Light Gradient Boosting Machine (lightGBM) is used to classify cancer types based on patient features, while an unsupervised clustering algorithm Density-Based Spatial Clustering of Applications with Noise (DBSCAN) is utilized to divide single cancer subtypes.

## Materials and methods

### Datasets

The DNA methylation data, mRNA expression data, somatic mutation data, and patient clinical data employed in the study are all downloaded from the TCGA database (Wang *et al.* 2016). We consider 14 cancer types with a total of 5,290 samples (details in Table 1). We collect 5 proven human protein–protein interactomes: (1) by combining 2 publicly available high-quality yeast-two-hybrid (Y2H) datasets, binary PPIs were investigated by high-throughput Y2H systems (Rolland *et al.* 2014; Luck *et al.* 2020); (2) BioPlex V2.016 data on protein complexes discovered by strong affinity purification mass spectrometry techniques (Huttlin *et al.* 2015); (3) Low-throughput or high-throughput experimental tests based on literature from KinomeNetworkX (Cheng *et al.* 2014), Human Protein Resource Database (Peri *et al.* 2004), DbPTM 3.0(Lu *et al.* 2013), PhosphoNetworks (Hu *et al.* 2014), and Phospho.ELM (Dinkel *et al.* 2011); (iv) low-throughput experiments from Signa-Link2.0 are used to create a signaling network (Fazekas *et al.* 2013); and (v) IntAct (Orchard *et al.* 2014), InnateDB (Breuer *et al.* 2013), and low-throughput tests based on literature or protein 3-dimensional structures from BioGRID (Chatr-Aryamontri *et al.* 2015). All genes correspond to Entrez ID and duplicate PPI pairs are removed.

**Table 1.** Fourteen cancer types and corresponding sample numbers.

Cancer types		Patient number
BLCA	Bladder urothelial carcinoma	406
BRCA	Breast invasive carcinoma	750
CESC	Cervical squamous cell carcinoma	302
COAD	Colon adenocarcinoma	278
HNSC	Head and neck squamous cell carcinoma	504
KIRC	Kidney renal clear cell carcinoma	263
LIHC	Liver hepatocellular carcinoma	369
LUAD	Lung adenocarcinoma	453
LUSC	Lung squamous cell carcinoma	366
READ	Rectum adenocarcinoma	91
SKCM	Stomach adenocarcinoma	468
STAD	Stomach adenocarcinoma	369
THCA	Thyroid carcinoma	498
UCEC	Uterine corpus endometrial carcinoma	173

**Fig. 1.**
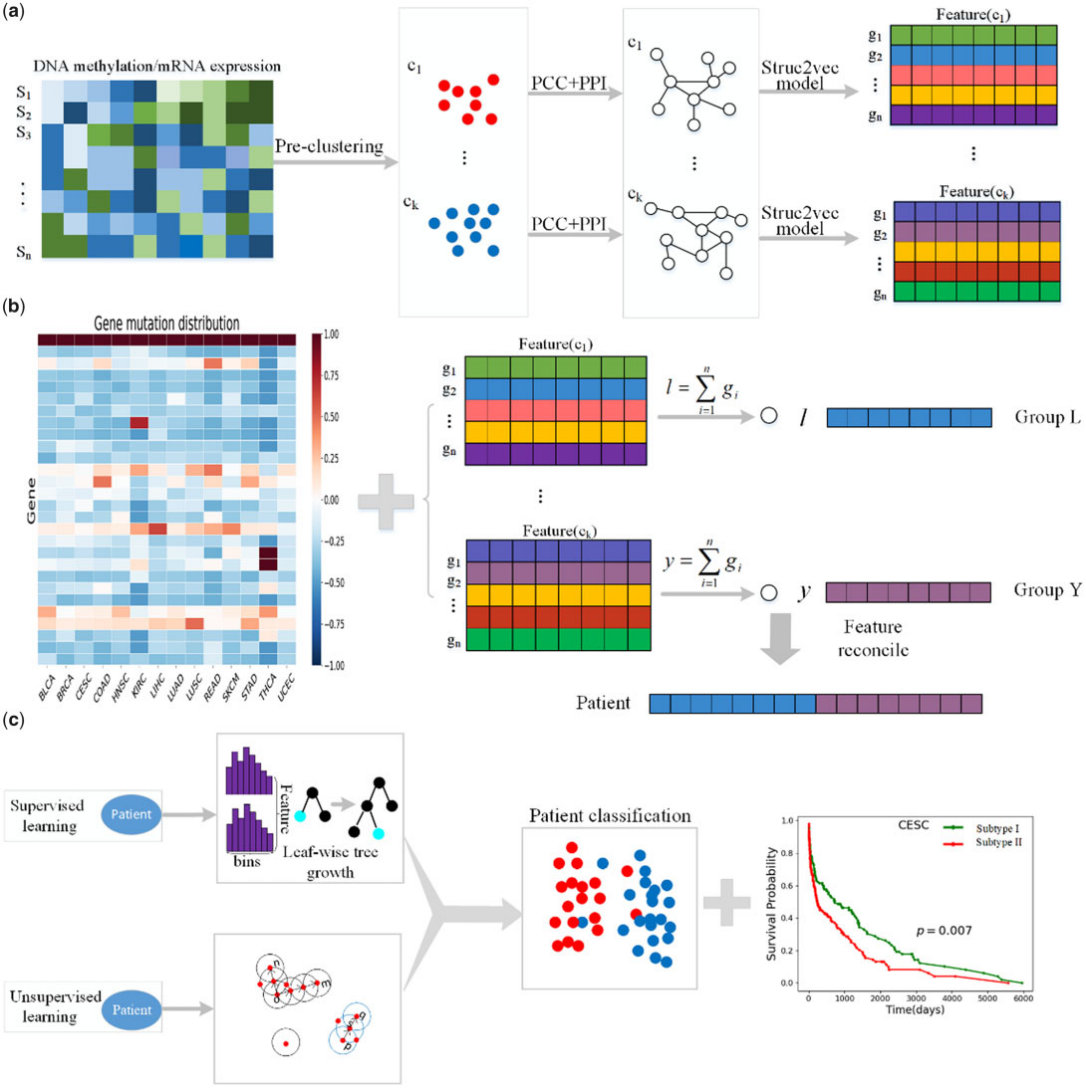
The overview of NESM.(a) Samples are divided into groups using DNA methylation and mRNA expression data. Pearson correlation is calculated among genes in the group. The gene pairs with strong correlation are then preserved in PPI. Next, the network embedding is performed using the struc2vec model. (b) The gene feature generates in step (a) are combined with the somatic mutation spectrum of the patient to construct the patient features. (c) The patients constructed in step (b) are partitioned using machine learning methods and verified by survival curves.


The overview of NESM is illustrated in Fig. 1. It consists of 3 parts: (1) Samples are clustered using DNA methylation and mRNA expression data. And the Pearson correlation between genes is calculated in each cluster. Then, the gene pairs with strong correlation are preserved in PPI. Next, the network embedding is performed using the struc2vec model. (2) The gene feature generates in step (1) are combined with the somatic mutation spectrum of patients to construct patient features. (3) Patients constructed in step (2) are divided using machine learning methods and validated by survival curves.

K

**Figure:**
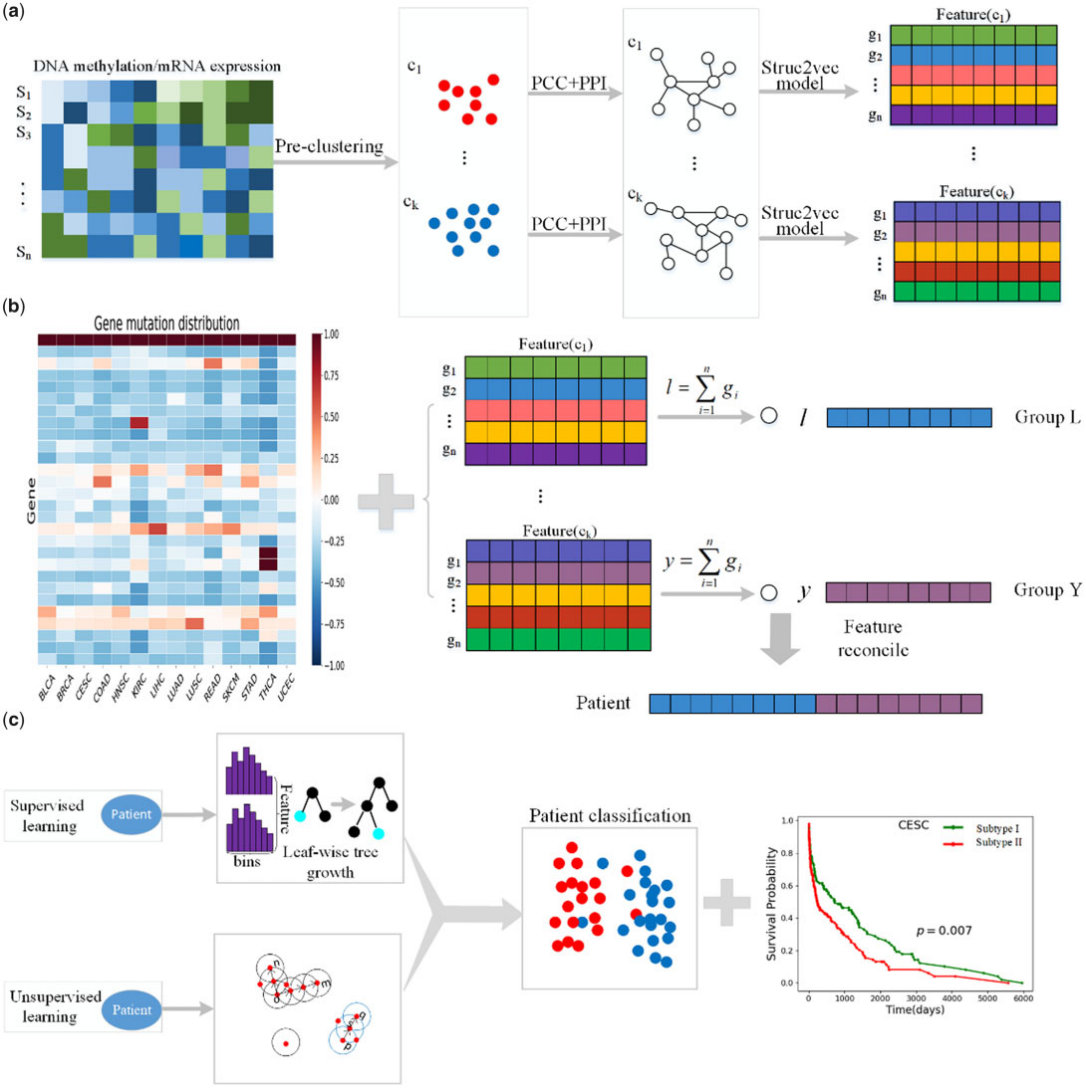


### Network embedding

The interactions between genes are reflected in the PPI network. To better mine the features of genes in the PPI network, we adopt the struc2vec model (Ribeiro *et al.* 2017) for the vectorization process of PPI network nodes. The Struc2vec model encodes structural similarity by constructing a multilayer graph and generates structural context for nodes. Compared with most algorithms, it can find distant but structurally similar gene pairs, which is more conducive to constructing similar genetic features of patients. The struc2vec model's primary steps are as follows:

#### Compute structural similarity

The structural similarity f(x,y)between each pair of nodes x and y can be denoted as:

(1) $$\begin{array}{c} f_{k}(x,y)=f_{k-1}(x,y)+g(s(R_{k}(x)),s(R_{k}(y))),\\ g(s(R_{k}(x)),s(R_{k}(y)))=\frac{\max(s(R_{k}(x)),s(R_{k}(y)))}{\min(s(R_{k}(x)),s(R_{k}(y)))}-1, \end{array}$$

where $R_{k}(x)$ represents the set of vertices with k(k≥0) distance from the vertex x,$s(R_{k}(x))$ represents the order sequence of vertex set $R_{k}(x)$, and $g(s(R_{k}(x)),s(R_{k}(y)))>0$is a function measuring the distance between order sequence $R_{k}(x)$and $R_{k}(y)$ and $f_{-1}=0$.

#### Construct a hierarchical weighted graph

The edge weights of 2 nodes in the same layer are defined as:

(2) $$w_{k}(x,y)=e^{-f_{k}(x,y)},k=0,1,\ldots k^{*},$$

where $k^{*}$denotes the original network's diameter.

The same nodes belonging to different levels are connected by directed edges. For a node in the layer k, it will be linked to a node corresponding in the layer (k−1)and layer (k+1). The edge weight is defined as:

(3) $$\begin{array}{c} w(x_{k},x_{k+1})=\text{log}(\Gamma_{k}(x)+e),\\ w(x_{k},x_{k-1})=1, \end{array}$$

where $\Gamma_{k}(x)$ is the number of edges at the k layer whose edge weight is greater than the average edge weight of the edge connected to .x.

#### Generate node sequence

We use the biased random walk to carry out random walk in the weighted multilayer graph. It is assumed that the walk takes place in the current layer with the probability of q and jumps to other layers with the probability of (1−q). If it is determined to walk in the current layer, let it be in the layer k, then the probability from node to node is defined as:

(4) $$p_{k}(x,y)=\frac{e^{-f_{k}(x,y)}}{Z_{k}(x)},$$

where $Z_{k}(x)=\sum_{y\neq x}e^{-f_{k}(x,y)}$is the normalized factor x in the *k*th layer. Through the random walk, each sampled node is more inclined to choose the node with high similarity to the current node structure. If switching to other layers, the probability of selecting (k−1)and (k+1)is defined as:

(5) $$\begin{array}{c} p_{k}(x_{k},x_{k-1})=\frac{w(x_{k},x_{k-1})}{w(x_{k},x_{k-1})+w(x_{k},x_{k+1})}\\ p_{k}(x_{k},x_{k+1})=1-p_{k}(x_{k},x_{k-1}) \end{array}$$

Each random walk sequence in this study has a length of 80 steps. In addition, for each node, create 20 random walk sequences (Fig. 1a). The Skip-Gram model (Mikolov *et al.* 2013) is applied to train node sequences while creating them. A 128-dimensional feature is contained in each gene.

### Constructing patient features

To better describe patients, we use mutated genes to construct patient features. We find that the frequency of genes mutations is different in different cancers. As illustrated in Fig. 1b, some genes are mutated in all cancers, while others are mutated only in certain cancers. Therefore, we define a weight to balance the effects of different genetic mutations. The weight is defined as:

(6) $$w(n)=\frac{v_{i}(n)}{u(n)},$$

where u(n)is the total number of gene nmutations in the 14 cancers, and $v_{i}(n)$ is the total number of gene nmutations in cancer $v_{i}$. We create a new 128-dimensional feature by fusing mutant genes from the same sample in the same cluster. Finally, the identical samples are spliced across all clusters to create a 1,280-dimensional vector that represents patient features.

### Supervised classification and unsupervised clustering models

The constructed patient features are classified using the lightGBM (Ke *et al.* 2017) classification method, which is a supervised approach based on Gradient Boosting Decision Tree (Chen and Guestrin 2016). When classifying cancer types, tumors with the same cancer are taken as positive samples and tumors with other cancers are taken as negative samples. For dichotomies, we use the AUC (Area Under Curve) value as the evaluation index of classification performance. AUC is the area under the Receiver Operating Characteristic (ROC) curve, which is an evaluation index to evaluate the merits of a dichotomous model.

We use DBSCAN clustering to cluster different subtypes of same cancer, which is an unsupervised density clustering method (Ester *et al.* 1996). Given the neighborhood radius δ, the threshold of the number of data objects in the neighborhood MinPts, for the cluster M (temporary), the domain of p(p∈N)can be calculated using the formula:

(7) $$N_{\delta }(p)=\left\{q\in M|d(p,q)\leq \delta \right\},$$

where the distance between the node p and qis denoted by d(p,q). If $N_{\delta }(p)\geq \mathrm{MinPts}$,p is the central point.

## Results

### Pan-cancer classification

**Fig. 2.**
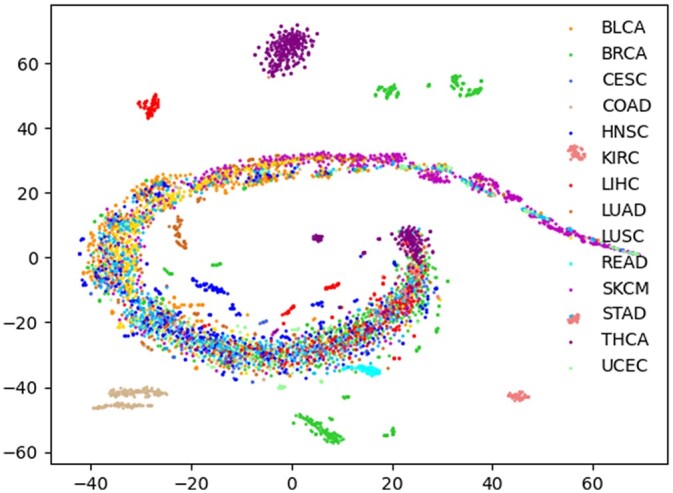
Visualization of patients using t-SNE.

In this work, we randomly choose 14 cancer types, but we are not limited to 14, from the TCGA database to collect the corresponding clinical information, mRNA expression data, DNA methylation data, and gene mutation data. We preprocess data for each cancer type by obtaining common samples containing all 3 data, normalizing the mRNA expression data and averaging the methylation sites on the same gene. Then, a total of 5,290 samples with gene mutation, DNA methylation, and mRNA expression data in 14 cancer types are obtained (details in Table 1). We generate features by integrating the list of mutated genes and the genetic features obtained through network embedding. Here, each patient is represented by a feature of 1,280 dimensions. To view the distribution status of 14 cancer patients, we visualize patients with 14 cancer types using the t-distributed stochastic neighbor embedding algorithm (Van der Maaten and Hinton 2008) and represented by different colors. We find that most patients of the same type tended to cluster together (Fig. 2). This is due to the fact that different cancer types have different mutation frequencies (Fig. 1b), and patients with the same cancer type are more likely to cluster together, while patients with different cancer types are separated.

**Fig. 3.**
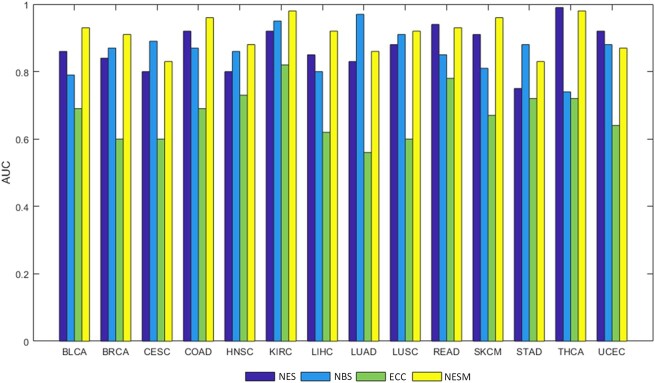
The AUC values of our NESM method are compared with those of ECC, NBS, and NES methods for 14 cancer types.

**Fig. 4.**
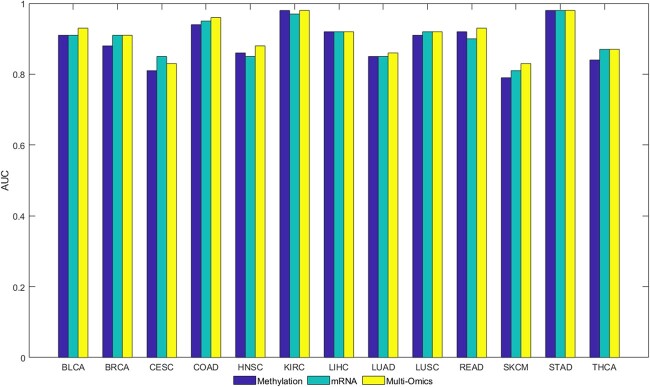
The AUC values are compared under the NESM framework using mRNA expression, methylation, and multi-omics data.

We use the lightGBM classification algorithm to predict the patient subpopulations and patient features as input of the algorithm to test the feasibility of NESM. When identifying cancer types, the corresponding cancer patients are positive samples, and other cancer patients are negative samples. In the case of colon adenocarcinoma (COAD), patients in COAD cancer are considered as positive samples, while patients from other cancers are negative samples. By using 5-fold cross validation, the positive and negative samples are separated into training and testing sets. The 5-fold cross validations perform 100 times and the average of the results is the final AUC value. Moreover, we compare with 3 latest methods: NES, NBS, and ECC methods. As shown in Fig. 3, our method has obvious advantages in bladder urothelial carcinoma (BLCA), breast invasive carcinoma (BRCA), and COAD cancer types. It is slightly lower than the NBS method in cervical squamous cell carcinoma (CESC) and lung adenocarcinoma (LUAD) cancer types and slightly lower than the NES method in uterine corpus endometrial carcinoma (UCEC) cancer. On the whole, our method is better than the other 3 methods. Furthermore, we show that using a single omics data for classification is less effective than using multi-omics fusion (Fig. 4).

### Stratification of specific cancer subgroups

**Fig. 5.**
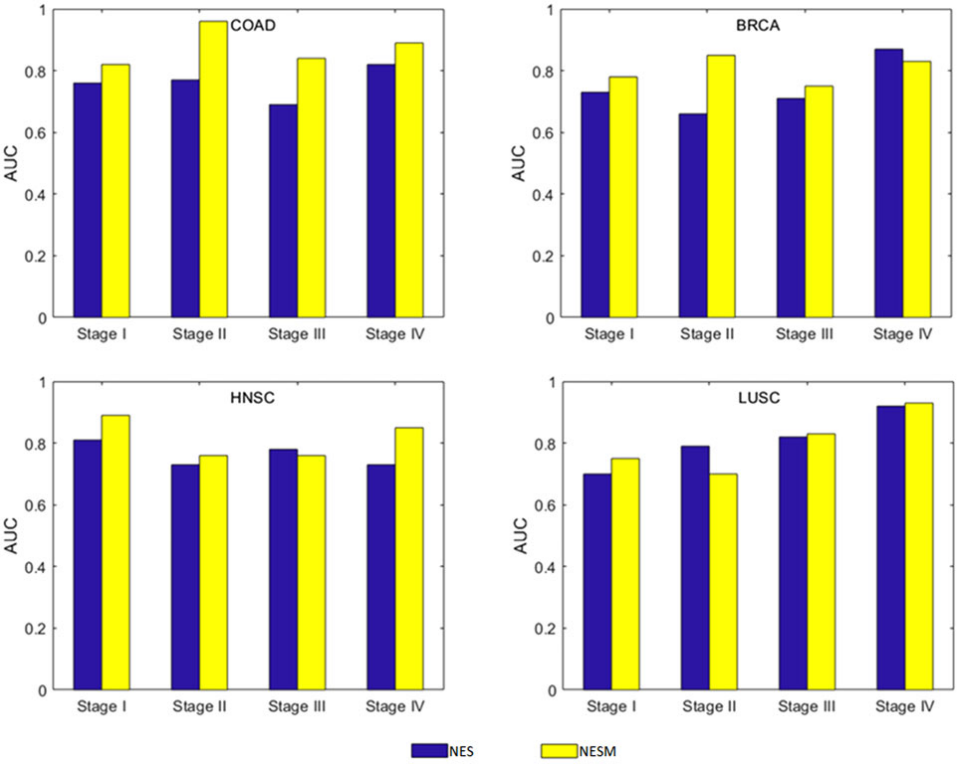
AUC of patients with NESM and NES tumor stages is compared in COAD, BRCA, HNSC, and LUSC cancer types.

Another aim in this work is to classify patients with the same cancer type into the corresponding subtypes. We assemble clinical information on patients with 14 cancer types from the TCGA database and obtain staging information to assess the stratification of tumor mutations. The tumor stage refers to the primary tumor and the extent of intraindividual spread. In general, the more extensive the spread, the worse the prognosis. The tumor-node-metastasis (TNM) staging system is the most widely utilized around the world. After T, N, and M are determined in TNM staging, corresponding general staging can be obtained, namely stage I, II, III, and IV. The stage I COAD tumor is taken as an example, and the stage I COAD tumor is labeled as a positive sample. Nonstage I COAD tumor markers are negative samples. We find that the average AUC of most tumor stages is higher than 0.75, while the average AUC of kidney renal clear cell carcinoma (KIRC) tumor stages is 0.66. Figure 5 shows our comparison with the NES method in COAD, BRCA, head and neck squamous cell carcinoma (HNSC), and lung squamous cell carcinoma (LUSC) data. Under COAD data, COAD outperforms NES in all cases. Under BRCA data, NESM is higher than NES at stages I, II, and III. Under HNSC data, NESM is higher than NES at stages I, II, and IV. Under LUSC data, NESM is higher than NES at stages I, III, and IV. In summary, our method has some advantages. (Supplementary Fig. 1 illustrates the staging results of 14 tumors.)

**Fig. 6.**
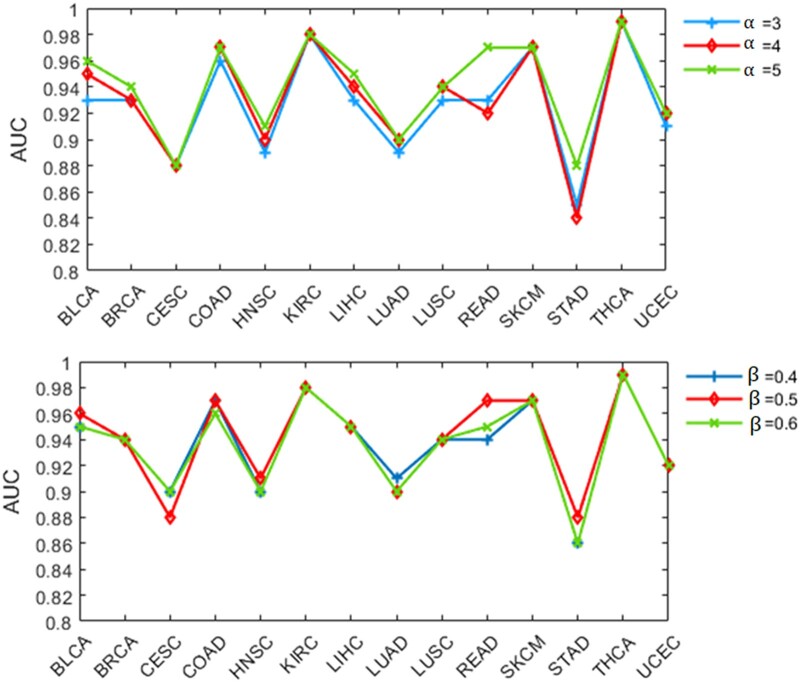
The line chart shows the effect of NESM on the classification of 14 cancers under different parameters.

To test the algorithm's robustness, we evaluate the parameters involved in the algorithm. In our method, 2 main parameters affect the algorithm: initial clustering and weight screening. We find that the time cost of the algorithm increases with the increase of parameter value. Therefore, we take α as 3, 4, and 5 and βas 0.4, 0.5, and 0.6, respectively, for discussion. When parameter αis 3, 4, and 5, the corresponding AUCs are 0.89, 0.90 and 0.91, respectively. When parameter βis 0.4, 0.5, and 0.6, the corresponding AUCs are 0.90, 0.91, and 0.90, respectively. The AUC values under different parameters are given in Fig. 6, and the small fluctuation range evidences that NESM is robust.

**Fig. 7.**
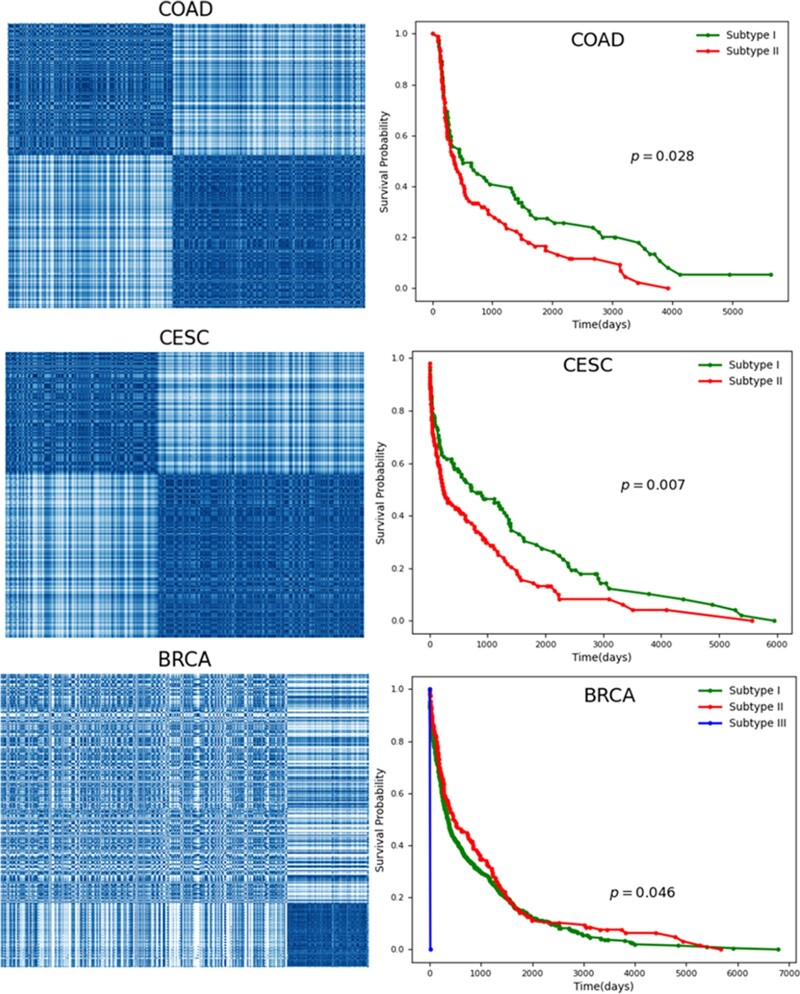
The results of tumor mutation stratification in COAD, CESC, and BRCA cancers. At the left, patient clusters are presented, with dense patches indicating that comparable individuals should cluster into the same subtype. Survival analysis of patients with different subtypes is shown on the right, and P-values are calculated based on the log-rank test.

Based on the above studies, we believe that patients with similar clinical information may be more inclined to cluster together. This means that we can obtain the optimal cluster of patients through an unsupervised learning algorithm, that is, patients are subdivided. We use the DBSCAN clustering algorithm to cluster the same cancer patients. The number of clusters matches the number of subtypes reported in the literature when it comes to identifying medical tumor subtypes. Taking CESC as an example, it is divided into 2 subtypes according to clinical and endocrine features (divided into—type I and type II) or histopathological features (divided into endometrioid, serous or clear cell adenocarcinoma). We generate 2 groups of patients with endometrial carcinoma (CESC) in the data. We also evaluate the clustering results by calculating the Silhouette Coefficient and Calinski–Harabasz Index (Supplementary Table 2). For the vast majority of cancer types, patients are closely distributed in the same population. Furthermore, we assess patient survival. As can be seen from the Kaplan–Meier survival diagram in Fig. 7, survival rates of the identified subtypes significantly differ from time and probability. For example, the survival times of the 2 CESC subtypes have noteworthy differences ($P=7\times 10^{-3}$). We provide COAD, CESC, and BRCA clustering results in Fig. 7 and carry out survival analysis for each subtype. The lower P-values indicate that the subtypes identified by NESM are reliable. In addition, we provide clustering and survival analyses (P<0.05) for other cancers in Supplementary Figs. 2 and 3. Across 14 cancer types, the majority of cancer subtypes identified by NESM are significantly associated with patient survival.

## Discussion

Cancer is a multifaceted illness caused by both hereditary and nongenetic components. With the development of technology, multi-omics data has recently been widely used for various cancer types. In this work, NESM constructs patient features by integrating corresponding gene features and somatic mutation profiles of cancer types. Since network topology information is extracted by integrating DNA methylation, mRNA expression data and PPIs through network embedding method, it is contained though the gene features. We apply supervised classification algorithms to classify pan-cancer and individual cancer stages. The experimental results show that the patient features extracted by the NESM method are effective for tumor stratification. When cancer subtypes are subdivided, the vast majority of subtypes identified by the NESM method are significantly associated with patient survival. NESM extracts features mainly from network topology, which is not considered by most methods.

It allows better classification and subdivision of cancers into subtypes than other methods, but it still has some limitations. For example, the choice or construction of a PPI network may have an impact on the NESM model. In addition, the rate of somatic mutation varies greatly among different tumor types. Some tumor types [such as stomach adenocarcinoma (STAD), UCEC, and others] have a high mutation rate, while others have a low mutation rate [such as rectum adenocarcinoma (READ) and BRCA]. In the current NESM framework, we only integrate normal tumor samples that match somatic mutation profiles, DNA methylation, and mRNA expression data. Integration of other types of omics data, including RNA sequencing, individual patient proteomics, and whole-genome sequencing, may further improve the NESM model. Second, the framework of the method is to cluster patients based on patient features extract from specific data sets. This framework can be used to address the tumor stratification problem using a variety of additional algorithms. For example, we can use a graph convolution neural network to improve the prediction accuracy and use other clustering algorithms, including hierarchical clustering and Gaussian mixture model clustering. In future work, it will provide some clues for precision oncology and clinical applications.

## Supplementary Material

jkac243_Supplemental_Material

jkac243_Figure_Supplemental_S1

jkac243_Figure_Supplemental_S2

jkac243_Figure_Supplemental_S3

## Data Availability

The code of NESM is available at https://github.com/FengLi12/NESM. Mutation data for PPI and BLCA, BRCA, CESC, COAD, HNSC, KIRC, liver hepatocellular carcinoma, LUAD, LUSC, READ, stomach adenocarcinoma, STAD, thyroid carcinoma, and UCEC are obtained from the literature: doi:10.1093/bioinformatics/btaa1099. DNA methylation and mRNA data are obtained from https://xenabrowser.net/datapages/. Supplemental material is available at G3 online.
