# Supplementary material for: NESM: a network embedding method for tumor stratification by integrating multi-omics data
Source: G3 (Bethesda). 2022 Sep 19;12(11):jkac243. doi: 10.1093/g3journal/jkac243 (PMC9635646; doi:10.1093/g3journal/jkac243)
Supplement: jkac243_Supplemental_Material [file jkac243_supplemental_material.docx]

Supplement to “NESM: A Network Embedding Method for Tumor Stratification by Integrating Multi-Omics Data”

Feng Li^1^, Zhensheng Sun^1^, Jin-Xing Liu^1^, Junliang Shang^1^, Xikui Liu^2^ and Yan Li^2^,*

1 School of Computer Science, Qufu Normal University, Rizhao 276826, China.

2 Department of Electrical Engineering and Information Technology, Shandong University of Science and Technology, Jinan, Shandong 250031, China

* Corresponding author: [liyanhd@163.com](mailto:liyanhd@163.com)

**Supplementary Materials**

**Figure S1.** The bar plot of the corresponding AUC of the patient classification for each tumor stage across 14 cancer types.

**Figure S2.** Visualization of the patients clustering for 14 cancer types. The dark blue area indicate that the corresponding patients should be clustered into the same subtype.

**Figure S3.** The survival analysis for NESM-predicted subtypes (clusters) across 14 cancer types.

**Table S1.** The key parameters for the proposed method.

| Network Embedding | |
| --- | --- |
| the number of the random walks start for each gene | 20 |
| the length of each random walk sequence | 80 |
| the number of layers | 6 |
| the context size for optimization | 5 |
| the dimension of the node vector | 128 |
| LightGBM | |
| boosting type | GBDT |
| number of leaves | 20 |
| max tree depth | 4 |
| learning rate | 0.01 |

**Table S2.** The Silhouette Coefficient and Calinski-Harabasz Index to evaluate the clustering results for each cancer types.

|  | Silhouette Coefficient | Calinski-Harabasz |
| --- | --- | --- |
| BLCA | -0.18 | 21.07 |
| BRCA | -0.31 | 10.26 |
| CESC | 0.39 | 89.15 |
| COAD | 0.29 | 56.10 |
| HNSC | 0.17 | 94.07 |
| KIRC | -0.03 | 12.22 |
| LIHC | 0.81 | 292.52 |
| LUAD | 0.27 | 189.85 |
| LUSC | 0.07 | 15.75 |
| READ | -0.01 | 3.09 |
| SKCM | 0.34 | 151.27 |
| STAD | 0.66 | 245.47 |
| THCA | 0.07 | 8.48 |
| UCEC | 0.41 | 47.48 |
