# Supplementary figures and images for: NESM: a network embedding method for tumor stratification by integrating multi-omics data
Source: G3 (Bethesda). 2022 Sep 19;12(11):jkac243. doi: 10.1093/g3journal/jkac243 (PMC9635646; doi:10.1093/g3journal/jkac243)

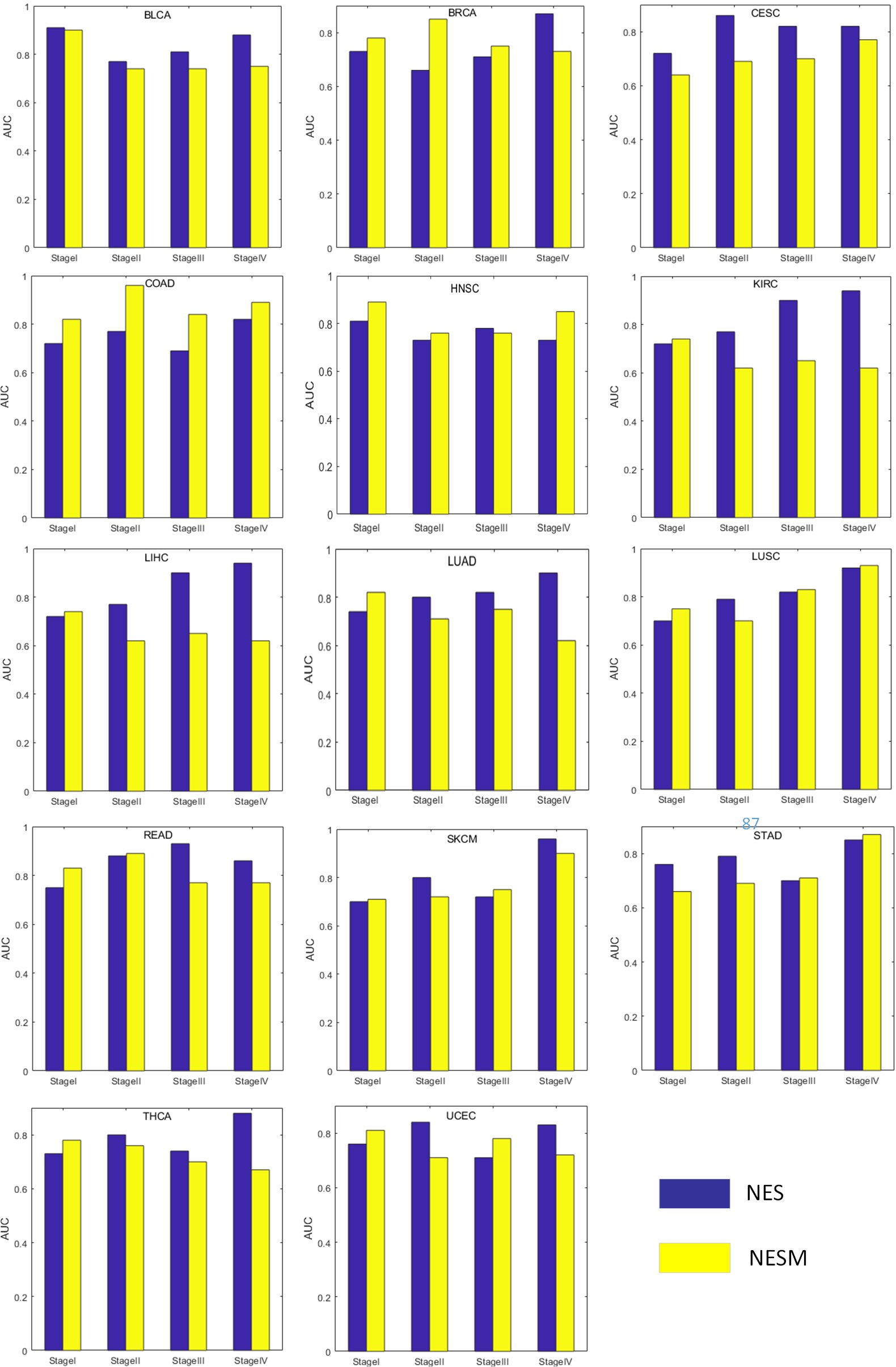

Supplement: jkac243_Figure_Supplemental_S1 [file jkac243_figure_supplemental_s1.jpeg]

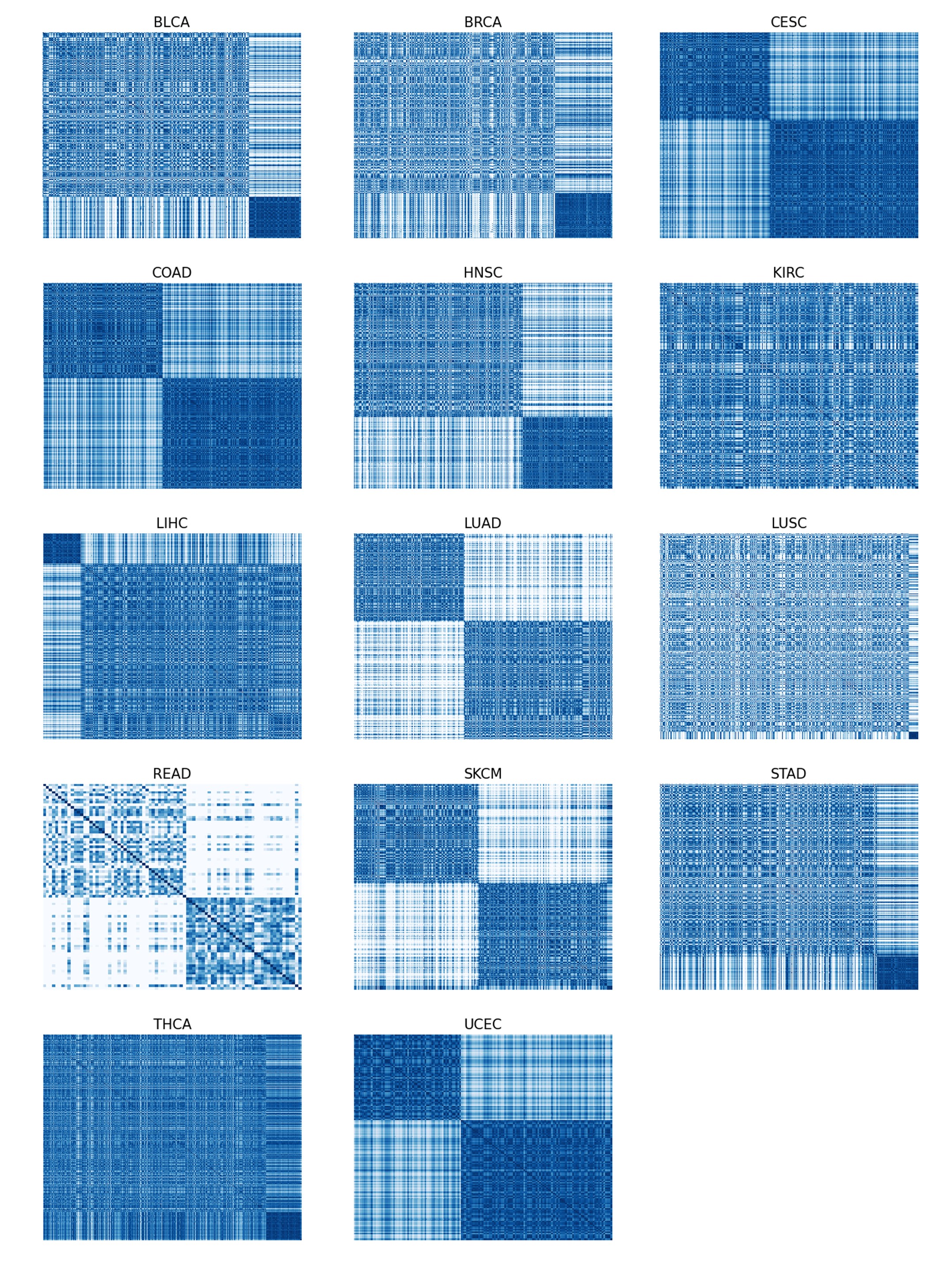

Supplement: jkac243_Figure_Supplemental_S2 [file jkac243_figure_supplemental_s2.jpeg]

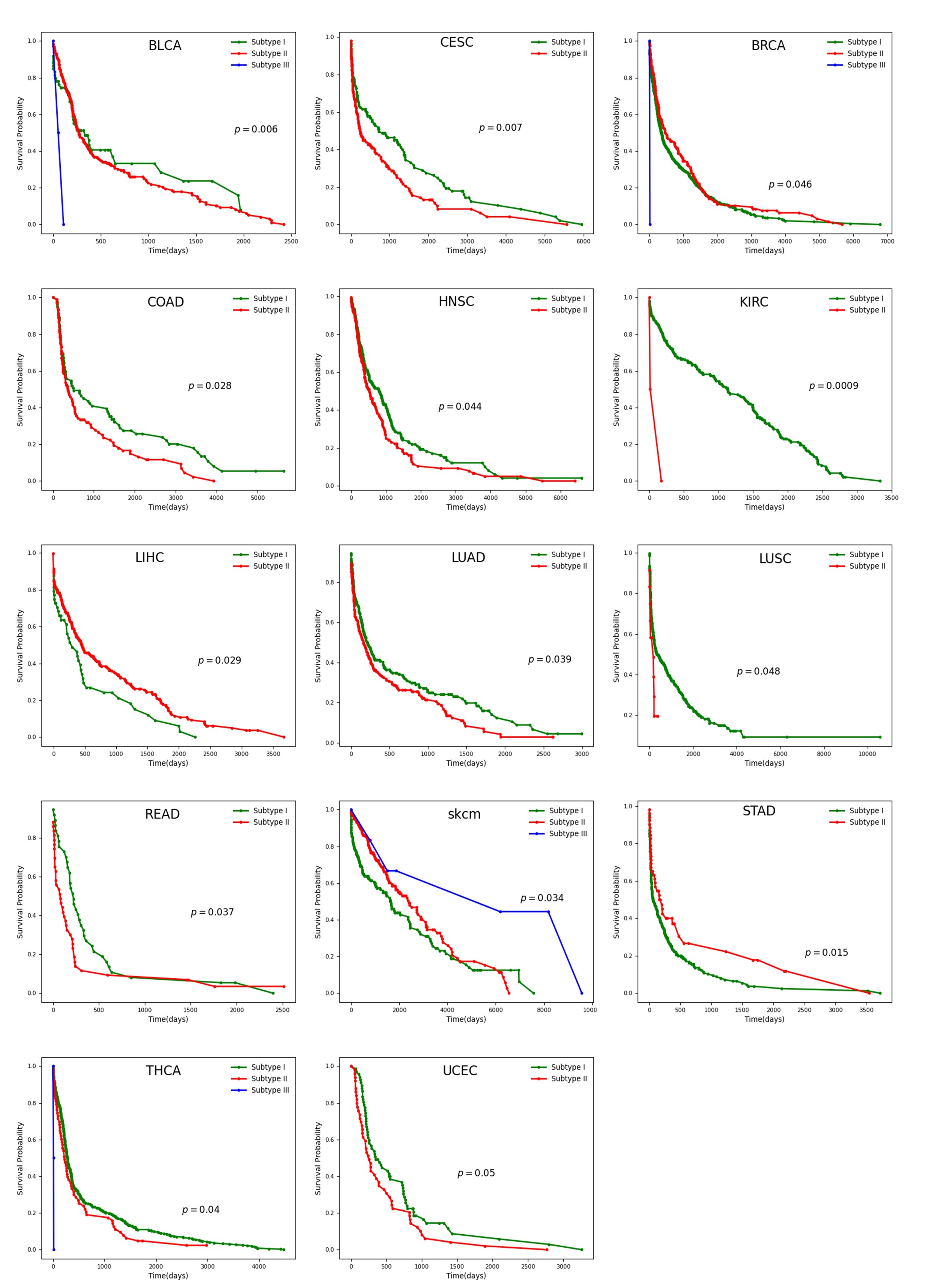

Supplement: jkac243_Figure_Supplemental_S3 [file jkac243_figure_supplemental_s3.jpeg]
